# Expected Competencies and Personal Attributes of Digital Health Navigators to Support Digital Mental Health Care: Focus Group and Interview Study With Patients and Health Care Professionals

**DOI:** 10.2196/83073

**Published:** 2026-04-23

**Authors:** Laura Freisberg, Eva Meier-Diedrich, Martin Heinze, Julia Schönbeck, Darja Schubert, Justin Speck, Gillian Strudwick, John Torous, Julian Schwarz

**Affiliations:** 1 Faculty of Health Sciences Brandenburg, Brandenburg Medical School Theodor Fontane, Neuruppin, Germany; 2 Department of Psychiatry and Psychotherapy, Immanuel Hospital Rüdersdorf, Brandenburg Medical School Theodor Fontane, Seebad 82/83, Rüdersdorf bei Berlin, 15562, Germany, 49 33638 83-501; 3 Centre for Addiction and Mental Health, Toronto, ON, Canada; 4 Institute of Health Policy, Management and Evaluation, University of Toronto, Toronto, ON, Canada; 5 Division of Digital Psychiatry, Beth Israel Deaconess Medical Center, Harvard Medical School, Boston, MA, United States

**Keywords:** digital mental health, digital therapeutics, DTx, Digitale Gesundheitsanwendungen, DiGA, competencies, digital health navigator

## Abstract

**Background:**

Digital mental health apps (DMHAs), and in particular digital therapeutics (DTx), offer promising opportunities to support mental health care. However, their effective use in outpatient settings in Germany remains limited. To overcome this gap, the role of digital health navigators (DHNs) has been introduced. DHNs are trained individuals who support patients and health care professionals in selecting, using, and integrating DMHAs into care. Despite increasing interest in this role, there is limited evidence on the competencies, knowledge, and personal attributes required for DHNs to work effectively in mental health settings.

**Objective:**

The study aims to explore the expected competencies, knowledge areas, and personal attributes that DHNs need to effectively support the implementation and use of DTx in outpatient mental health care.

**Methods:**

As part of the prestudy of the Digital Navigators for Acceptance and Competence Development with Mental Health Apps (DigiNavi) study, a qualitative study was conducted involving 35 participants (7 general practitioners, 8 patients in general practice, 11 outpatient psychiatrists/psychologists, and 9 patients in psychiatric outpatient clinics) from different general practices and psychiatric outpatient clinics in Germany. A total of 17 semistructured interviews and 4 focus groups were conducted to explore expectations of DHNs. Data were analyzed using qualitative content analysis.

**Results:**

Participants emphasized that DHNs should combine strong interpersonal skills (empathy, patience, and sensitive communication) with technical and basic clinical competencies. Most favored DHNs as integrated clinical team members (eg, medical assistants), citing their existing patient relationships, but noted time and training constraints. Key expectations included the ability to support patients with DTx use, adapt communication to individual needs, and convey data privacy information clearly. Foundational knowledge of mental health conditions and sensitivity to crises were considered important for identifying warning signs and escalating concerns. While DHNs were seen as essential intermediaries between patients, health care professionals, and DTx, participants highlighted the necessity for clearly defined roles, structured training, and realistic expectations to prevent role overload and enable sustainable implementation in outpatient mental health care.

**Conclusions:**

DHNs require a specialized skill set that bridges clinical understanding, digital expertise, and interpersonal competence. Our results lay the groundwork for developing training curricula and implementation strategies that align with real-world expectations for the DHN role. Defining these core competencies is essential for supporting the sustainable and effective integration of DMHAs into mental health care.

## Introduction

Mental health conditions are a significant global concern, with a substantial and increasing number of individuals affected [1,2]. A recent study shows that approximately half of the global population is expected to experience at least one mental disorder by the age of 75 years [3]. This rising prevalence has profound implications not only for individuals but also for societies and economies worldwide.

The burden of poor mental health extends far beyond the direct costs of treatment, encompassing substantial indirect costs associated with reduced productivity. Depression and anxiety disorders alone cost the global economy approximately US $1 trillion annually in lost productivity [4]. In contrast, the total economic impact of mental health conditions, including lower employment rates and reduced productivity, is estimated at up to 4.2% of GDP in Organisation for Economic Co-operation and Development countries [2]. Furthermore, untreated mental health conditions often follow a chronic course and are associated with substantially increased risks of physical comorbidities, excess premature mortality, and greater health care usage [5,6]. Addressing mental health issues promptly is thus essential to prevent long-term consequences. Early intervention and access to appropriate care can mitigate the progression of mental disorders [7].

However, access to psychological treatment remains limited, particularly in low- and middle-income countries due to structural barriers [4]. High-income countries face similar access constraints, with insufficient treatment capacity resulting in prolonged waiting times [8,9]. Access to psychotherapy is even more limited for individuals living in rural areas, where service availability is typically lower, and geographical barriers further restrict timely care [10]. For individuals in rural areas, general practitioners (GPs) are often the first point of contact for mental health problems. However, even if the mental disorder is recognized and diagnosed, GPs frequently lack the time, specialized training, and structural capacity to provide guideline-concordant treatment [11,12].

One opportunity, in addition to the long-term expansion of psychiatric and psychotherapeutic care, is the integration of digital mental health apps (DMHAs) into health care. Among these, digital therapeutics (DTx) represent a specific category of DMHAs. They are software-driven, evidence-based interventions that can be prescribed or recommended by clinicians to prevent, manage, or treat mental health conditions [13]. As such, DTx holds particular promise for bridging the persistent treatment gap by helping patients maintain or improve their mental health [14,15]. Furthermore, DTx can significantly improve accessibility for individuals in remote or underserved regions, particularly those who face marginalization [16]. New regulatory frameworks for DTx have been introduced worldwide [17]. In Germany, the so-called “Digitale Gesundheitsanwendungen (DiGA; in English: digital health application)” refers to low-risk, Conformité Européenne–marked digital health apps that are intended for the diagnosis, monitoring, or treatment of diseases. They must demonstrate positive health effects to be reimbursed by statutory health insurance. Germany implemented legislation for DiGA in December 2019, becoming the first country in the world to combine regulatory approval with reimbursement for DTx [18,19]. Since then, approved DTx have been reimbursed by statutory health insurance funds.

However, widespread adoption remains limited in Germany [20,21]. Several key factors contribute to this [20,22], including the fact that many health care professionals (HCPs), particularly physicians, lack sufficient knowledge and awareness of DTx, their benefits, and effective implementation strategies. Time constraints faced by HCPs also pose a challenge, limiting their capacity for patient onboarding and explaining DTx usage. The complexities of integrating DTx into existing health care workflows, alongside concerns surrounding data security and patient privacy, represent additional barriers to their widespread adoption [23]. Furthermore, low patient adherence and engagement significantly hinder the successful integration of DTx [24], as well as low digital literacy among patients and HCPs [25-27].

**Figure 1.**
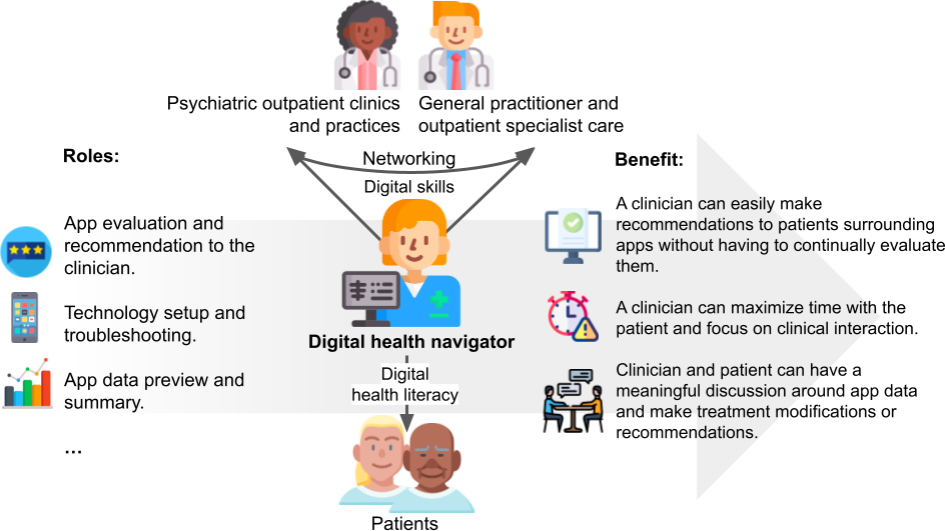
Role and tasks of digital health navigators.

To address these challenges, emerging international models have introduced the role of digital health navigators (DHNs) [28-33]. DHNs are specialized HCPs working in inpatient or outpatient care, tasked with facilitating the informed, effective use of DMHAs within routine care [34]. Early experiences from countries such as the United States suggest that DHNs can play a critical role in supporting patients and clinical teams alike. By offering technical assistance, providing information on the clinical relevance of DTx, and fostering motivation and engagement, DHNs may enhance the implementation of DTx in mental health services [35]. According to Wisniewski et al [35], DHNs fulfill at least 3 core functions (refer to Figure 1, which is adapted from their conceptualization): first, they maintain knowledge on available mental health apps, evaluate them based on evidence, and support both HCPs in selecting appropriate mental health apps and patients in using them. Second, DHNs train the multidisciplinary care team in basic technical and digital skills and provide ongoing support, including troubleshooting for technical problems. Third, they process app-generated patient data and prepare it in a clinically useful format for HCPs. However, the successful integration of this new role depends heavily on the specific competencies and attributes that DHNs bring to the clinical environment [35]. In addition to technical proficiency and knowledge of available DMHAs, interpersonal skills, clinical sensitivity, and an understanding of data privacy concerns are considered crucial [34]. Recent implementation studies have further specified these requirements: Chen et al [31] presented a detailed manual for DHN implementation in a cognitive behavioral therapy–focused clinic, emphasizing training as a primary challenge, while Wang et al [36] identified patience, cultural humility, and lived community experience as key characteristics of successful health technology navigators in a US safety-net system.

However, as the DHN role is new and not yet formally defined in Germany, it is unclear which responsibilities and competencies stakeholders actually associate with this emerging function. Role theory provides a useful conceptual background for examining how expectations toward new professional roles take shape when responsibilities and boundaries have not yet been formally established [37]. It highlights that insufficient role clarity or inconsistent expectations, often described as role ambiguity and role conflict, can hinder coordination and complicate the integration of new roles into existing health care structures [38,39]. Applying role theory to the emerging DHN role, therefore, provides a useful lens for examining which competencies stakeholders consider necessary and whether these expectations are realistic and compatible.

Against this background, and given the lack of a standardized DHN role or training in Germany, this study systematically explores stakeholder expectations regarding the necessary competencies for DHNs within the German health care context [40]. Understanding the skill sets and personal characteristics deemed important by patients and HCPs can inform the development of targeted training programs and contribute to the effective and sustainable implementation of DHNs.

Although existing studies have described DHN tasks, implementation frameworks, and general competency areas [31,34-36,41], systematic research on the specific skills and credentials DHNs require remains limited. Pape et al [33] explicitly identified this gap, noting the need for research to help health care organizations understand “the requisite credentials or skills a digital health navigator must have.” Addressing this gap in the German context, our study examines stakeholder expectations to inform a clearer definition of the DHN role and the development of training programs aligned with health care system requirements. To provide a focused analytical frame, we formulated the following primary research question: How do patients and HCPs conceptualize the competencies required for the emerging DHN role in outpatient mental health care? Through iterative qualitative analysis, three core domains emerged that structure our findings: (1) role requirements, (2) soft skills (interpersonal, communicative, and work style competencies), and (3) hard skills (technical, digital, and clinical knowledge).

## Methods

### Study Design

This prestudy is part of the overarching DigiNavi (Digital Navigators for Acceptance and Competence Development with Mental Health Apps, Medical School Brandenburg, July 15, 2024, to September 30, 2025) project that investigates both the acceptance and engagement of DHNs in German outpatient care [40,42]. Within the DigiNavi study, DHNs are selected and trained to support patients in using DTx for mental health over a period of 3 months. This qualitative prestudy was developed in accordance with the COREQ (Consolidated Criteria for Reporting Qualitative Research) checklist (Checklist 1) [43] and examined the acceptance and expectations of DHNs from the perspective of patients and HCPs. This paper focuses on expected core competencies, knowledge areas, and personal attributes of DHNs. Given the exploratory nature of this study, a qualitative design was chosen to ensure a comprehensive and thorough examination of the expected DHN characteristics [44,45]. The study was guided by one overarching research question. Initial deductive categories were derived from the interview and focus group guides, which had been developed before data collection, based on existing literature. During the iterative coding process, these categories were continuously refined, expanded, and reorganized inductively. Through this process, 3 overarching analytical domains (role requirements, soft skills, and hard skills) gradually emerged as the final thematic structure of the findings.

### Participants

In total, 35 individuals from 6 study centers (3 general practices and 3 psychiatric outpatient clinics) in rural and urban areas of Brandenburg participated in the qualitative prestudy. The sample included 18 HCPs and 17 patients.

Among HCPs, 7 were GPs, and 11 were outpatient psychiatrists or psychologists. Among patients, 8 were recruited from general practices and 9 from psychiatric outpatient clinics. A balanced gender ratio was aimed for, and additional effort was made to include marginalized groups in the German health care system to address inequalities in access to digital mental health care [46].

Data collection involved 17 semistructured interviews and 4 focus groups. Interviews were conducted with 4 GPs, 4 outpatient psychiatrists/psychologists, 3 patients in general practice, and 6 patients in psychiatric outpatient clinics. Focus groups included 1 group with patients in general practice (n=5), 1 with patients with psychiatric conditions (n=3), 1 with GPs (n=5; 2 of whom had also participated in interviews), and 1 with outpatient psychiatrists/psychologists (n=7).

### Recruitment

Patients and HCPs were recruited through regional study centers in the federal state of Brandenburg, including general practices and psychiatric outpatient clinics. These centers were directly approached via email by the research team. Additionally, HCPs were recruited through professional networks. Recruitment of patients followed a gatekeeper approach, which implies that the study centers identified and invited suitable patients. Interested participants could then register for participation by contacting a member of the study team via email. Attempts to recruit via the Brandenburg Practice-Based Research Network (FoPraNet-BB) through email distribution and a project presentation at a research congress did not yield usable responses. Supplementary recruitment strategies included public outreach via social media and the dissemination of study-specific flyers through cooperating study centers. Interested participants received an information sheet detailing the study purpose, procedures, duration, and time compensation (€25 [US $28.45] for patients and €50 [US $56.90] for HCPs).

Eligible participants for this study (1) were aged ≥18 years, (2) were able to give informed consent, (3) had access to a modern digital device (eg, smartphone) to participate in a digital survey, (4) had sufficient language skills to complete an interview in German, (5) were not at acute risk of danger to themselves or others, (6) had no severe organic brain disorders with cognitive impairments, and (7) were not intellectually disabled (IQ-based or clinically diagnosed).

### Data Collection

Before the interviews and focus groups, all participants completed a web-based sociodemographic questionnaire (Multimedia Appendix 1), which was hosted by the online survey service LimeSurvey Professional (LimeSurvey GmbH) [47]. Gender and ethnicity were assessed via self-report using predefined categories in the sociodemographic questionnaire; sex assigned at birth was not collected. Ethnicity was collected for descriptive purposes only, recognizing that it is a social construct. The interview and focus group guidelines (Multimedia Appendix 2) were developed using a mixed deductive-inductive approach. First, knowledge gaps were identified through a literature review and discussed within the team. Second, questions were formulated to address these gaps and capture unexpected perspectives. They focused on competencies and characteristics that patients and professionals consider crucial for potential DHNs. The semistructured guidelines balanced orientation toward the research questions with flexibility to explore relevant aspects [48]. No pretesting was conducted.

Interviews and focus groups were conducted between August 2024 and February 2025. The majority of interviews (13/17) were conducted by a female academic researcher (DS); the remaining 4 were conducted by a trained student assistant. All focus groups were moderated by a female academic researcher (DS). She was supported by a female academic researcher in 2 sessions (Julia Schönbeck and LF, one each) and by a trained student assistant in the remaining 2. With participant consent, all sessions were audio recorded, pseudonymized, and transcribed by the online transcription service f4x. Due to time constraints, transcripts were not returned to participants for comments or corrections. During the focus groups, 1 researcher was responsible for taking field notes to document both observations and contextual information [49].

Interviews took place online via the video conferencing platform Cisco Webex (Cisco Systems, Inc) [50] or by telephone, depending on participant preference, and lasted between 30 and 60 minutes. Focus groups with GPs took place online (1/4, 25%); the others were conducted at the study centers or GP practices (3/4, 75%), lasting 60‐105 minutes.

### Reflexivity

DS conducted the interviews after receiving training in qualitative research methodology. The coauthors had experience in qualitative and mixed methods research. All researchers had a background in psychology or medicine. There was no prior relationship established between DS and the participants. Efforts to establish a good rapport with the participants were made throughout the study. The interviews were individually adapted to the flow of discussion of each participant. DS also shared her research interests with the participants after the interviews and focus groups (debriefing).

### Data Analysis

Demographic data were analyzed descriptively using Stata/MP (version 17; StataCorp LLC). Qualitative data were analyzed by the primary data collector (DS) in MAXQDA (VERBI Software GmbH) following Kuckartz qualitative content analysis [45]. DS familiarized herself with the material through partial transcription, transcript verification, and repeated review of all recordings before coding. At the outset, a provisional set of deductive categories was derived from the interview and focus group guides. These guides had been developed based on existing literature on DiGAs and their implementation in routine care, including physician and patient perspectives, barriers to adoption and adherence, and supportive and navigator-based models in digital health [35,41,51-53]. These served as a starting point and were progressively reshaped and extended inductively during coding. The 3 analytical domains (role requirements, soft skills, and hard skills) gradually consolidated during this iterative process and represent the final analytical structure. DS piloted the coding frame on approximately 60% of the data. DS and LF independently coded overlapping subsets of the material and met regularly (7‐14-day intervals) to compare coding decisions, discuss divergences, and refine category boundaries. Following this, DS presented the provisional coding frame in a qualitative research workshop (University of Ulm) and integrated external feedback into the analytic structure. The finalized frame was then applied to the complete dataset. Because our focus was on thematic content rather than interactional dynamics, interview and focus group data were treated as a single analytic corpus. Both formats followed the same semistructured guideline and yielded comparable thematic content. Analyzing both data sources jointly allowed the identification of competency expectations that were articulated consistently across individual and group-based settings and across professional and patient perspectives. This approach supported a cross-contextual understanding of the DHN role rather than prioritizing interactional dynamics within a single format. Nevertheless, the joint analysis might result in a loss of method-specific information, particularly concerning interactional dynamics unique to focus groups and the greater depth of individual interviews. Moreover, potential differences between data types might be lost when analyzed together. Thematic saturation was reached after the 35th participant, at which point no new themes or relevant variations emerged in subsequent transcripts [54]. A framework analysis matrix was subsequently used to compare patterns across participant groups and consolidate categories. Illustrative quotes were translated into English using DeepL (DeepL SE) [55] and verified by the team. Role theory served as an interpretive lens during the discussion of findings.

### Ethical Considerations

The study was approved by the Ethics Committee of the Brandenburg Medical School (MHB; reference no 218062024-BO-E) and registered at the German Clinical Trials Registry (DRKS; DRKS00034327), and at ClinicalTrials.gov (NCT06575582). All participants were informed about the study procedures (both verbally and in writing) as well as the possibility to withdraw their consent at any given time. Written informed consent was obtained from all participants. All data collected were pseudonymized to safeguard participant information. Patients received €25 (US $28.45), and HCPs received €50 (US $56.90) as compensation for their time. The ethics committee reviewed and approved the compensation as an appropriate reimbursement.

## Results

### Overview

**Figure 2.**
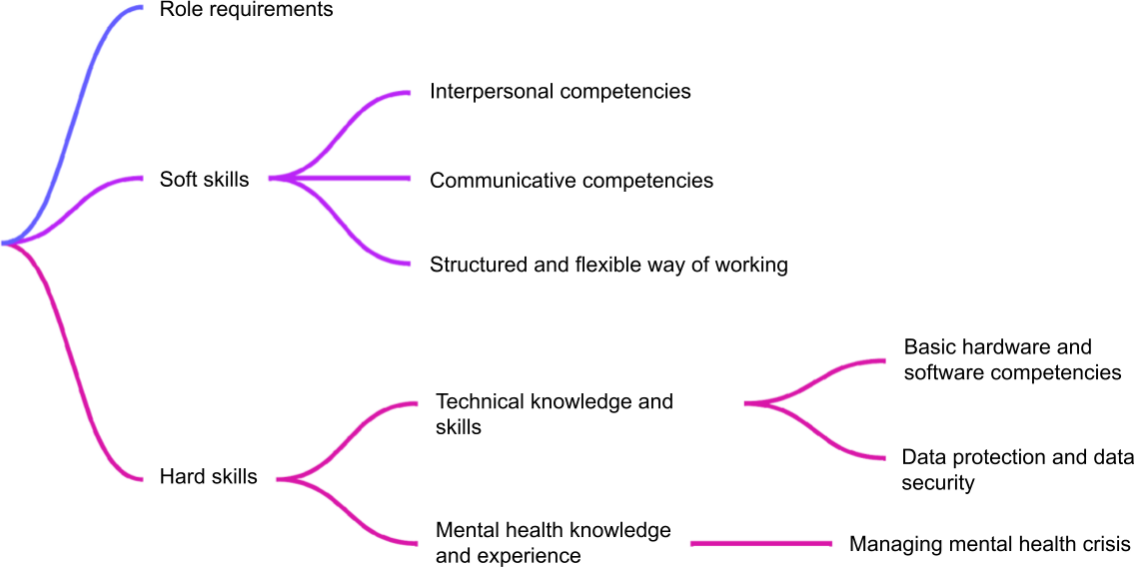
Thematic structure of expected competencies, knowledge, and personal attributes of digital health navigators (DHNs) in mental health care, as identified through qualitative content analysis.

Through iterative analysis, the findings were consolidated into 3 overarching domains (refer to Figure 2). First, participants described the professional and sociodemographic characteristics that DHNs should possess. Second, they identified the specific soft and hard skills considered essential for the role.

### Sample Characteristics

A total of 35 individuals participated in the study, including 17 (48.57%) patients and 18 (51.43%) HCPs from the participating study centers. Among the 17 patients, 9 (52.94%) received psychiatric care, and 8 (47.06%) were seen in the GP. Among the 18 HCPs, 7 (38.88%) worked in general practice and 11 (61.11%) in psychiatric settings. Detailed sociodemographic information on the individual participants, their roles, and key sociodemographic features is provided in Table 1 (patients) and Table 2 (HCPs).

**Table 1.** Sociodemographic details of participating patients (N=17).

Characteristic and category	Value
Setting, n (%)	
Psychiatric care	9 (52.94)
Male	6 (35.29)
General practice	8 (47.06)
Age (years), mean (SD; range)	45.3 (17.42; 22‐72)
Sex, n (%)	
Female	11 (64.70)
Ethnicity, n (%)	
White	16 (94.12)
Person of color	1 (5.88)
Education, n (%)	
Elementary or main school	1 (5.88)
Secondary school	4 (23.53)
High school diploma	4 (23.53)
Vocational training	4 (23.53)
University degree	4 (23.53)
Employment status, n (%)	
Full time	3 (17.64)
Part time	2 (11.76)
Unemployed	1 (5.88)
Retired	6 (35.29)
Unable to work	5 (29.41)
Current or prior psychotherapeutic treatment	12 (70.59)

**Table 2.** Sociodemographic details of participating health care professionals (N=18).

Characteristic and category	Value
Setting, n (%)	
Psychiatric care	11 (61.11)
Age (in years), mean (SD; range)	48.78 (10.78; 26‐65)
General practice	7 (38.88)
Sex, n (%)	
Female	13 (72.22)
Male	5 (27.77)
Ethnicity, n (%)	
White	16 (88.88)
Other	2 (11.11)
Professional roles, n (%)	
Medical specialist	6 (33.33)
Medical practitioners in training	5 (27.77)
Licensed psychotherapist	3 (16.66)
Psychotherapist in training	1 (5.55)
Social worker	2 (11.11)
Professional experience (years), mean (SD; range)	20.38 (10.45; 0.8‐36)

In relation to their prior knowledge about DTx, most patients indicated having no (7/17, 41.18%), very little (5/17, 29.41%), or rather little knowledge (4/17, 23.52%). Only a minority had prior experience with DTx: about one-third (6/17, 35.29%) had ever used a well-being app, and fewer than one-quarter (4/17, 23.52%) had ever received a DTx prescription. Regarding their confidence in using digital applications, patients’ responses were almost evenly distributed: approximately one-third each reported feeling rather insecure (5/17, 29.41%), rather secure (5/17, 29.41%), or highly secure (6/17, 35.29%). Around half of the patients rated their ability to learn how to use new digital applications as either rather low (8/17, 47.06%) or high to very high (7/17, 41.17%). When encountering technical difficulties, patients most commonly sought help from others or attempted to resolve the issues independently (7/17, 41.17% in each case). Most participating HCPs rated their prior knowledge of DTx as low (15/18, 83.33%). Slightly more than half (10/18, 55.55%) had ever been prescribed a DTX. However, the majority reported feeling confident (6/18, 33.33%) or very confident (8/18, 44.44%) in using digital applications, and most assessed their ability to learn new digital tools as relatively high (15/18, 83.33%). Given the small and ethnically homogeneous sample, no analysis stratified by ethnicity was conducted.

### Qualitative Results

The following section presents an integrated summary of the qualitative findings derived from the interviews and focus groups. Where frequencies are reported, they are intended to illustrate the range and recurrence of perspectives across the sample rather than to imply quantitative weighting or prevalence. Key participant quotes were selected to illustrate each thematic category and are generally displayed in a table at the end of each section. Each quote is followed by the corresponding participant identifier (eg, PP00) and role designation (HCP [GP], HCP [psych], patient [GP], and patient [psych]). The letters of the identifier indicate the study center, while the numbers refer to the individual participant.

### Role Requirements

In the interviews and focus groups, participants discussed the role requirements for DHNs. DHNs were described as an “intermediary between the patient and the health care service” [PP08, patient], and the qualitative data revealed diverse yet complementary perspectives regarding the most suitable professional background, language skills, and age of DHNs. Some participants (4/35, 11.42%) advocated for DHNs to be members of the existing clinical team. They emphasized that the long-standing relationships between patients and clinical staff could foster trust and help reduce barriers to engagement. In contrast, other HCPs (3/35, 8.57%) suggested that the DHN role could also be fulfilled by external individuals, such as peer supporters. In both cases, participants underscored that the successful integration of DHNs into the treatment concept would require reliable and long-term structural anchoring.

With regard to the occupational group best suited for the DHN role, participants proposed and discussed various options. Some (8/35, 22.86%) identified medical assistants, nurses, and physician assistants as particularly appropriate candidates, as these professionals are often long-term employees within clinics or practices, possess relevant medical and organizational knowledge, and maintain close relationships with patients. At the same time, several participants (4/35, 11.42%) expressed doubts about the suitability of these groups, arguing that their training might not sufficiently cover the communicative and therapeutic competencies required for the DHN role. Consequently, social workers, psychologists, and psychotherapists (3/35, 8.57%) were also mentioned as potentially suitable candidates due to their more extensive therapeutic training. Irrespective of the occupational background, participants (6/35, 17.14%) generally agreed that the DHN role entails additional responsibilities. Given that medical assistants, nurses, and physician assistants often operate within tightly scheduled clinical routines, some participants (3/35, 8.57%) pointed out the need to carefully consider who within a team actually has the capacity to take on these tasks. Feasibility and workload constraints were thus identified as important practical boundaries that must be considered when defining the scope of DHN responsibilities.

A few participants (2/35, 5.71%) further highlighted that DHNs should be fluent in the national language and ideally proficient in additional languages (at least English) to overcome potential language barriers and enable unrestricted communication with patients. Opinions on the ideal age of DHNs varied: while 1 (2.86%) HCP regarded age as irrelevant, others (3/35, 8.57%) favored younger DHNs, assuming they might be more digitally adept and better able to engage with the target population. Across these categories, participants consistently emphasized that DHNs should approach their tasks with motivation and genuine interest and be able to establish a good rapport with patients. It became evident that certain barriers could be reduced simply through the choice of person filling the DHN role—for instance, through existing familiarity and trust, or through multilingual abilities (refer to Table 3 for illustrative quotes).

**Table 3.** Sample quotes for the role requirements for digital health navigators.

Topic	Quotes
Integration in the medical team	“[The DHN]^a^ should be a team member, I would say. But it’s clear to me that if we’re doing this here at our site, then it should be someone from the team who wants to do it, who simply says: I want to get involved, I take the responsibility.” (SM07, HCP^b^ [psych^c^]) “Now that I think about it, I could even imagine that it doesn’t necessarily have to be a doctor or something, but perhaps even a former patient.” (RP01, patient [psych])
Occupational group	
Medical assistants	“I believe that they really need to be medical professionals who have an interest in having contact with patients.” (RM03, HCP [psych]) “I also don’t believe that a medical assistant is in a position to conduct this conversation. They are simply not trained for it.” (TM01, HCP [GP^d^]) “And secondly, she [the medical assistant] simply doesn’t have the time. Even if she becomes a well-trained DHN, and I see this as more of a time problem.” (TM01, HCP [GP])
Psychologist, psychotherapist, and social worker	“Ideally, I would say, it should be persons who already have experience in that respect, who have experience with people in general, perhaps social workers or psychologists or I don’t know, or in any case also have training.” (RP08, patient [psych])
Language	“I think it would be helpful if they spoke fluent English – or another language. It would be good if they were a bit multilingual, especially since we do have a lot of international patients.” (BM03, HCP [GP])
Age	“Because what I’ve asked our team [who will take the DHN role], is that most of them are younger people who may be in training and are interested in it.” (EM01, HCP [GP]) “I wouldn’t necessarily pin it [the DHN] down to a certain age.” (SP02, patient [psych])

a DHN: digital health navigator.

b HCP: health care professional.

c psych: psychiatric outpatient clinic.

d GP: general practice.

### Soft Skills

#### Interpersonal Competencies

A key prerequisite for a successful implementation of the DHN role appears to be the interpersonal competencies of the DHN. This also includes the ability to establish a trusting relationship with patients that is characterized by mutual trust and openness (5/35, 14.28%). Many participants (11/35, 31.43%) emphasized that the DHN-patient relationship should not be limited to technical support but should be marked by interpersonal warmth and empathy. It was considered the DHN’s task to establish this kind of emotional connection by engaging with patients in a way that is emotionally resonant and genuinely interested. In addition to empathy, DHNs should adopt a consistently understanding and patient attitude, according to some participants (8/35, 22.86%). Patience, openness, composure, and a calm demeanor were described as essential characteristics.

Participants considered it particularly important to avoid communication that could be perceived as intrusive or coercive. This includes, on the one hand, the willingness to repeatedly explain digital or technical processes without becoming irritated or condescending (8/35, 22.86%), and, on the other hand, recognizing and respecting patients’ individual limits, which may be related to their illness, and refraining from pressuring them to use DTx or the DHN service (7/35, 20%). Instead, DHNs should seek to sensitively understand the reasons behind noncompliance.

#### Communicative Competencies

Closely connected to interpersonal competencies are the communication skills expected of DHNs. Participants (11/35, 31.43%) emphasized that DHNs should communicate with patients in a professional yet sensitive manner, showing appreciation, empathy, and mutual respect. A key part of this is actively listening to patients and taking their perspectives seriously. In addition, DHNs should be able to explain medical and technical content in a lay-friendly way (4/35, 11.43%). During appointments, DHNs should adopt an open, curious, and supportive attitude toward patients, free of judgment or condescension, and maintain a nondirective stance (5/35, 14.29%). Moreover, participants (3/35, 8.57%) highlighted the importance of DHNs being able to stimulate patients’ interest in DTx and convey the benefits of such applications in a motivating way. This style of communication should reflect the principles of motivational interviewing, gently encouraging patients while maintaining a supportive tone.

#### Structured and Flexible Way of Working

Several participants (5/35, 14.29%) emphasized the importance of a certain level of autonomy in the DHN’s role. Once the HCP has prescribed the DTx, DHNs are expected to independently arrange regular follow-up appointments with both patients and HCPs, providing opportunities for case discussion. The interviewees highlighted the need for DHNs to establish clear structures and routines that align with existing practice workflows. This included regular coordination and communication with the practice team. Thus, DHNs were expected to provide consistent and reliable support for patients according to a well-defined process and to be capable of managing billing-related tasks where applicable.

Given that DHNs interact with a wide range of patients, participants (6/35, 17.14%) considered adaptability and flexibility essential in addition to a well-structured approach. DHNs should be able to adjust their communication style and mode of interaction (eg, via phone, video call, or in-person visit) to suit individual patient needs. They should remain responsive to patients’ diverse life circumstances, including their digital and technical literacy, and tailor their support accordingly. Together, these findings highlight the central role of interpersonal, communicative, and organizational competencies for effective DHN performance (refer to Table 4 for illustrative quotes).

**Table 4.** Sample quotes for the soft skills for the digital health navigators.

Topic	Quotes
Interpersonal competencies	“So the conversational aspect—I think that would be really important from my perspective, that the DHN^a^ not only has digital skills but can also truly build this interpersonal relationship.” (BM01, HCP^b^ [GP^c^]) “It can be helpful to establish a basis of trust for the conversations or to discuss things. And not become overarching, so to speak, lecturing.” (RP07, patient [psych^d^]) “Of course, also take the time to listen to individual wishes and, yes, just make sure that care is as patient-centered as possible.” (PP08, patient [GP])
Communicative competencies	“And yes, just appropriate communication. So that it still remains professional and nonjudgmental, and that you really communicate, act, and refer on in the interest of the patient.” (PP08, patient [GP]) “And in terms of skills, having a friendly approach with patients and also, I’d say, sticking with it, motivating them, being persistent, like: ‘I’ll call you again.’” (FM01, HCP [psych]) “Personally, I would value medical competence—being able to explain the background to me in a way I can understand.” (PP06, patient [GP])
Structure and flexibility	“I would clearly expect that the DHN, that this person can integrate into existing workflows in such a way that they check when we have team meetings and identify time slots when we might be able to sit down together, to ask questions or coordinate when and how things can be scheduled.” (SM06, HCP [psych]) “It would also be great, just like the medical assistants do in everyday practice, if they kept track of their activities themselves. Meaning they would record the initial prescription, document follow-up appointments, and consistently keep an eye on the billing codes relevant to DiGA^e^.” (BM01, HCP [GP]) “They should be able to adapt. If someone needs more support or lacks knowledge, they should be able to respond to that. But also, for someone like me with a high level of education, they should adjust accordingly.” (RP08, patient [psych])

a DHN: digital health navigator.

b HCP: health care professional.

c GP: general practice.

d psych: psychiatric outpatient clinic.

e DiGA: Digitale Gesundheitsanwendung.

### Hard Skills

#### Technical Knowledge and Skills

In addition to interpersonal and communicative abilities, DHNs require technical and digital knowledge. However, from the patients’ perspective (3/35, 8.57%), it appears particularly crucial that digital-technical knowledge is combined with interpersonal and communicative skills, as illustrated by the following quote: “The person should really have at least decent technical skills and be able to deal with people. Those, I think, are the most important things.” [PP06, patient]

According to many interviewed participants (15/35, 42.86%), DHNs should possess fundamental digital skills and be proficient in using common digital devices. Having a general affinity for technology was considered a key prerequisite for the role. DHNs should be able to operate computers, tablets, and smartphones; be familiar with common operating systems and web applications; and, most importantly, be capable of installing, setting up, and using health apps. Several interviewees (5/35, 14.29%) stated that DHNs should be aware of existing DTx and keep up with current developments in the field to support patients in choosing, installing, and configuring DTx. DHNs are also expected to competently and clearly convey digital knowledge to patients when needed (3/35, 8.57%) and to assist them with more fundamental technical hurdles that may precede DTx use (eg, setting up an email account; 3/35, 8.57%).

#### Data Protection and Data Security

The DHNs’ technical and digital knowledge should be supplemented by a basic understanding of data protection, data security, data processing, and access rights relevant to DTx, according to the study participants (10/35, 28.57%). DHNs should be able to communicate these aspects clearly and comprehensibly to patients to ensure transparency and informed consent. In addition, DHNs should educate patients about how patient data are handled internally within clinics and practices while emphasizing that their own role is bound by professional confidentiality. Especially in the context of sensitive topics such as mental health, which are often associated with stigma, this was considered crucial for fostering patient trust. Some participants (2/35, 5.71%) also suggested that DHNs should receive advanced training in digital data protection, as existing internal training in clinics or practices often does not sufficiently address the specific requirements of digital environments. Such training would enable DHNs to act safely and competently in digital contexts and to pass this knowledge on to patients.

#### Mental Health Knowledge and Experience

In addition to digital competencies and knowledge of DTx, the interviewees (10/35, 28.57%) emphasized the importance of equipping DHNs with basic knowledge of mental health conditions and the ability to manage mental health crises. DHNs should be familiar with common (psychiatric) disorders and their typical symptoms. This basic knowledge seems essential for two main reasons: (1) improving communication and empathy with patients and (2) ensuring the correct DTx are selected. Other participants (4/35, 11.43%) acknowledged that, while some medical knowledge could be beneficial, it is not necessarily an essential requirement. They argued that many core DHN tasks, such as supporting patients in setting up and using DTx, can be performed effectively without in-depth medical knowledge, particularly if DHNs work in close collaboration with the treating clinicians. In addition to theoretical knowledge, practical clinical experience with patients with mental health conditions was considered valuable (7/35; 20%), as it can deepen theoretical understanding and enable DHNs to better adapt to patients’ individual needs.

Patients with mental health conditions are usually under general or psychiatric care and are regularly seen by clinicians. Nevertheless, it was considered important for DHNs to be able to recognize early warning signs of psychological deterioration and potential risk factors for a crisis (eg, progressing social isolation; 6/35, 17.14%). Participants emphasized that DHNs should be able to assess both gradually emerging problems and acute mental health crises, such as suicidality, psychosis, or delusional thinking, promptly and accurately. This includes taking appropriate de-escalating actions, such as involving the treating clinician, to prevent further decompensation.

In acute mental health crises, DHNs should respond to patients with empathy and emotional sensitivity. Furthermore, some participants (6/35, 17.14%) suggested that DHNs could offer a stabilizing point of contact in crises, primarily by recognizing early warning signs, responding with emotional sensitivity, and ensuring timely escalation to qualified clinicians. These expectations reflect perceived needs from a stakeholder perspective and do not imply that DHNs should assume clinical or therapeutic responsibilities in crisis management. Other participants (4/35, 11.43%) stressed the need for clear role boundaries and solid training for DHNs in managing such situations, emphasizing that clinical decisions and crisis interventions should remain the responsibility of qualified clinicians. It was considered essential that DHNs recognize their own limits (3/35, 8.57%), are not left to handle high-stakes situations alone, and maintain close communication with responsible clinicians. A critical concern was raised regarding whether DHNs could realistically be trained to manage such challenging situations. Compared to somatic illnesses, recognizing early signs of psychiatric conditions, which often emerge subtly, was considered significantly more complex.

Overall, these accounts point to a multifaceted competency profile that spans technical, data-related, and basic clinical domains (refer to Table 5 for illustrative quotes).

**Table 5.** Sample quotes for the hard skills for the digital health navigators.

Topic	Quotes
Technical knowledge and skills	
Basic hardware and software competencies	“They have to be able to handle smartphones, their operating systems, and also work with desktop computers, which have different operating systems again, and then also use web-based applications. So this concerns the technical domain.” (BM01, HCP^a^ [GP^b^])
DTx^c^ knowledge	“Well, they require skills, such as knowing the many apps and the mental disorders. Or, conversely, to know the mental disorder so well that the apps can be recommended. I think that’s a huge challenge because the apps are constantly changing.” (RNM01, HCP [GP])
Data protection and data security	“The person should at least be able to explain how the data is handled. For example, where the data is stored, what it is used for, those basic things. And I believe that’s also legally required. Of course, the app must present this information anyway, but it would be good if the person could explain it as well. Because people usually don’t read that stuff.” (PP06, patient [GP]) “That data protection is upheld in the way it’s regulated, and that the information doesn’t reach any outsiders, but stays between doctors, DHNs^d^, and the respective digital health application and doesn’t go beyond that. That way, patients can feel safe. Especially in mental health care, these are vulnerable topics and populations. And in that context, I think it’s particularly important that data protection is strictly observed.” (PP08, patient [GP])
Mental health knowledge, experience, and crisis management	“Well, the required competencies are knowing the different apps and the illnesses. Or rather, knowing the illnesses well enough to be able to recommend the right apps.” (RNM01, HCP [GP]) “I’m not even sure whether medical knowledge is really all that important. If questions come up, they can always discuss them with the treating clinicians. That way, things can be clarified. Of course, it doesn’t hurt to have some background, but I don’t think it’s a prerequisite for supporting patients, as long as someone with responsibility is in the background.” (RM03, HCP [psych^e^]) “Of course, there should be a basic level of knowledge, like if a patient starts expressing delusional ideas about the DHN, the alarm bells should ring and they should think, oh no, what’s going on? Maybe talk to the doctor quickly.” (RM02, HCP [psych])

a HCP: health care professional.

b GP: general practice.

c DTx: digital therapeutics.

d DHN: digital health navigator.

e psych: psychiatric outpatient clinic.

## Discussion

### Principal Findings

This qualitative study is the first to examine how patients and HCPs conceptualize the emerging DHN role, addressing 3 domains: role requirements, soft skills, and hard skills. Our findings highlight the broad and multifaceted expectations patients and HCPs have for this emerging role. Participants emphasized that DHNs must not only be technically proficient and clinically knowledgeable but also possess strong interpersonal and communicative competencies. The data point to a relatively demanding role profile for DHNs, characterized by wide-ranging and partially overlapping expectations at the interface between technical support and clinical responsibilities. From a role-theoretical perspective, this pattern illustrates risks of role ambiguity and role conflict as described in the literature [37-39].

A key finding is the strong emphasis on interpersonal and emotional competencies. These competencies were considered foundational for establishing trust and fostering engagement, a finding consistent with prior DHN research emphasizing the importance of soft skills for effective patient support [34,35]. Many participants highlighted that DHNs should not only support patients in using DTx but should also convey empathy, patience, and a nonjudgmental attitude. The ability to build rapport, listen actively, and communicate in a patient-centered and respectful manner was seen as essential. This includes being able to adapt to patients’ emotional states, digital literacy levels, and personal life situations.

In parallel, DHNs were expected to possess a relatively high level of technical and digital health literacy, including familiarity with smartphones and operating systems, troubleshooting skills, and knowledge about mental health DTx, including their evidence base, effectiveness, and methodological basis. Data protection literacy was considered particularly important in mental health care, where concerns around privacy and stigma are pronounced. Participants expected DHNs not only to understand data protection regulations related to DTx but also to communicate them clearly to patients and assist with app-specific settings if needed. These expectations align with core DHN functions described in the literature, including training, technical support, and digital health literacy education [33,35]. However, participants attributed additional responsibilities to DHNs, particularly regarding basic clinical sensitivity. While DHNs were not expected to replace psychotherapists, participants emphasized the importance of detecting early warning signs of mental health crises and escalating concerns appropriately.

Another recurring theme concerned the importance of flexible integration into existing care workflows. Participants discussed different professional backgrounds as potentially suitable for the DHN role, most frequently identifying medical assistants, psychologists, or nurses due to their familiarity with patients and practice procedures. At the same time, concerns were raised about limited time resources and existing workload pressures, particularly for medical assistants. Participants also pointed to the potential suitability of other professional or nonprofessional groups, such as peer supporters or community health care workers, depending on the health system context. Together, these accounts highlight feasibility constraints and the importance of role clarity when integrating DHNs into outpatient care.

Overall, our findings align with international DHN research while also revealing context-specific nuances. The competency areas identified mirror the 4 core functions described by Pape et al [33] (training, digital health literacy education, patient engagement, and workflow optimization) and the soft skills (patience and cultural humility) identified by Wang et al [36]. Consistent with the systematic review by Perret et al [41], our results highlight substantial heterogeneity in how the DHN role is conceptualized across settings. Notably, in the context of outpatient mental health care, our participants attributed a broader range of tasks to DHNs, including recognizing early signs of mental health crises, which extends beyond responsibilities typically defined in the literature [34,41]. While prior studies focused primarily on implementation experiences in North American contexts [31,33,36], our study contributes insights into stakeholder expectations before DHN implementation, thereby providing insights into how the role is anticipated and what competencies are considered essential within the German outpatient mental health care system.

Building on these empirical findings, several critical questions for implementation emerge: (1) How can the role of DHNs and the scope of their responsibilities be defined in a way that avoids overburdening them? (2) How can DHNs be smoothly integrated into the existing workflows of outpatient clinics and practices? (3) How can essential soft skills be effectively trained and maintained over time, given their central importance for patient engagement and trust? Ultimately, this points to a fundamental challenge: how to design the role of DHNs in a way that is both feasible in everyday clinical practice and beneficial for both health care teams and patients.

### Implications

The breadth of stakeholder expectations described in the “Results” section reflects the multifaceted needs within digital mental health care. The following considerations represent an interpretive assessment by the authors rather than a direct extension of stakeholder views. We argue that it is necessary to examine whether DHNs can realistically be expected to possess all the mentioned competencies. If medical assistants indeed carry out the duties of DHNs, we contend that the expectation voiced by some stakeholders—that DHNs should manage acute mental health crises—would exceed a realistic scope for this role. In Germany, diagnosis and management of mental health crises clearly lie within the responsibilities of qualified HCPs such as GPs, psychiatrists, or psychotherapists [56]. Medical assistants usually provide basic clinical and administrative assistance in outpatient care [57] and already face high demands in terms of workload, time pressure, and job intensity [58]. At the same time, participants’ comments also point to the potential suitability of other professional or even nonprofessional groups, such as peer supporters or community health care workers, particularly in contexts where medical assistants are not available or are structured differently. In light of these considerations, future implementation strategies will need to account for local workforce structures and care models. Given the discrepancies between the broad expectations voiced by stakeholders and the realistic limits of what DHNs can assume, our findings underscore the necessity of defining the scope of the DHN role in a way that prevents incompatible role demands and supports feasible implementation.

This distinction may be particularly important in psychiatric outpatient settings, where patients often present with more severe or unstable mental health conditions compared to general practice [59]. In such contexts, the symptoms are more complex, and the potential for acute symptom escalation or crisis is higher, which may unintentionally lead to an overextension of the DHN role if boundaries are not clearly defined. While DHNs may contribute valuable support in facilitating the use of DMHAs and monitoring usage patterns, we suggest that they should not be expected to handle clinical- or crisis-related responsibilities in psychiatric care settings. This does not preclude DHNs in any outpatient setting from detecting, identifying, and reporting signs of clinical deterioration to the treating professionals. However, their role should not be conflated with that of crisis responders, and we argue that clear boundaries regarding their responsibilities are essential to ensure both patient safety and role clarity.

Regardless of which professional group ultimately takes on the DHN role, participants emphasized the necessity of specific training to prepare individuals for the DHN function. Given the varying levels of digital, clinical, and communicative competencies across different health care professions, our analysis indicates that a standardized training curriculum may be necessary to ensure role clarity and quality of care. Such curricula may also need to be adapted to the specific professional backgrounds and health system contexts, recognizing that the optimal training pathway might differ across countries and settings. Based on our findings, we propose that effective DHN training should integrate both interpersonal (soft) skills and digital competencies, as these are closely intertwined in enabling patient-centered support and safe, competent use of DMHAs. Digital skills, in particular, are increasingly recognized as a core competency in modern health care and have been included in various international competency frameworks, such as the World Health Organization’s (WHO’s) Global Strategy on Digital Health [60]. However, studies show that many HCPs lack the required digital health literacy, especially regarding the use of DMHAs in routine care [61,62]. From an implementation perspective, this suggests that DHN-related training should not only address role-specific tasks but may also need to respond to the broader digital skill gaps within health care teams. Training approaches such as supervised practice, role-playing, reflective exercises, and ongoing feedback may offer feasible ways to foster and sustain these competencies. Future research should examine which training formats and content are most effective across different care settings.

A further challenge concerns the practical implementation of the DHN role, particularly with regard to embedding it into routine care processes without placing additional burden on existing health care staff. While the potential benefits of DHNs were acknowledged, participants also raised concerns regarding increased workload and unclear task distribution. Future implementation efforts should therefore investigate strategies for embedding the DHN role into existing workflows and team structures in ways that support acceptance and long-term feasibility. This may include clarifying collaboration between DHNs and other professional roles, allocating sufficient resources, and avoiding role overlap. Given that participants frequently identified medical assistants as suitable candidates for the DHN role, one potential implementation pathway could be to embed DHN training into existing vocational education programs, such as the training of medical assistants. In Germany, established training formats such as further modular qualification for medical assistants, enabling them to take over delegated tasks in primary care, offer a suitable framework for this integration [63]. As an illustrative example, within the broader DigiNavi project, a pilot refresher course conducted in collaboration with the Federal State of Brandenburg’s Medical Association was used to pilot the integration and reception of DHN-relevant content, such as DTx knowledge and patient-centered communication. Future work may examine whether and how such content could be incorporated more systematically into standard curricula.

Although these points extend beyond the empirical scope of our data, situating our findings within the broader digital mental health landscape helps to highlight potential system-level implications. Recent roadmaps position DHNs as central to equitable digital mental health implementation [64], while emerging frameworks increasingly address the evaluation of artificial intelligence–driven mental health tools [65]. Yet as digital solutions gain prominence, there is a risk that DMHAs and the DHN role could be instrumentalized as cost-effective substitutes for psychotherapy rather than as complementary elements within comprehensive care. Future policy efforts should ensure that the DHN role is integrated meaningfully into multiprofessional care and not used to compensate for structural underprovision of qualified psychotherapeutic services.

### Limitations

The study has several limitations. It reflects stakeholder expectations in a hypothetical setting because DHNs were not yet implemented in the participants’ care environments. This implies that findings are based on anticipatory perceptions rather than lived experiences. Furthermore, although the concept of DHNs may hold promise for use in inpatient care or somatic health care settings, the findings of this study are not readily transferable, as the focus was limited to mental health in outpatient contexts. Regarding recruitment, the involvement of study centers in identifying and enrolling participants may introduce a gatekeeping bias [66].

Moreover, since the study was conducted in a single, rural region (Brandenburg, Germany), caution is warranted when interpreting these findings beyond similar contexts. Several sample characteristics limit generalizability. First, the lack of ethnic diversity in our sample (94.12% of patients and 88.88% of HCPs self-identified as White) does not reflect the demographic composition of Germany’s urban centers, where a substantial proportion of the population has a migration background. Given that language barriers, access to care, and experiences with the health care system are shaped by social and structural factors that often correlate with ethnicity, stakeholder expectations regarding DHNs, particularly concerning language skills and cultural sensitivity, may vary considerably in more diverse settings. Second, digital literacy levels, access to technology, and familiarity with DiGAs may differ substantially between rural and urban populations. Participants in metropolitan areas may have higher baseline digital competencies, potentially leading to different expectations regarding the technical support functions of DHNs. Third, the structure and availability of mental health services vary between rural and urban regions in Germany. In Brandenburg, where access to specialized psychiatric care is often limited, participants may have emphasized certain DHN functions (eg, bridging care gaps) that would be less salient in well-resourced urban settings. Future research should therefore include samples from metropolitan areas and ensure adequate representation of ethnically diverse populations to examine whether the competency expectations identified here apply across different health care contexts.

Additionally, due to the convenience sample, the findings may not be representative of the entire population of HCPs and patients. A potential self-selection bias should be considered, as participation was voluntary and therefore participants who are more digitally interested may be overrepresented. Furthermore, patients who chose to participate in the interviews and focus groups may have been more motivated and less severely affected by mental illness than the average patient, potentially leading to differing expectations and needs. Regarding the analysis, interview, and focus-group data were treated as a single corpus given our focus on thematic content rather than interactional processes. While this approach was appropriate for our research question, it does not capture group-specific dynamics such as agreement or negotiation among participants. Accordingly, our findings emphasize shared thematic expectations rather than differences in how such expectations were negotiated within group discussions. Future studies explicitly focusing on the social construction of role expectations could therefore differentiate between these data sources to explore such interactional aspects in greater depth. More research is needed to examine the role of DHNs in real-world clinical settings to validate or refine the expectations identified here and to include samples from metropolitan areas and ethnically diverse populations to assess generalizability across different health care contexts. The applicability of the DHN role in other settings, such as inpatient care or somatic health care, also warrants investigation.

### Conclusions

The findings of this study underline the need for a clearly defined and realistic scope of responsibilities that aligns with existing structures in outpatient and inpatient care. In this context, structured and standardized training is essential to ensure that DHNs are adequately prepared for their tasks, can act confidently within their scope of responsibility, and contribute effectively to interprofessional health care. Without adequate preparation, there is a risk of role ambiguity, compromised care quality, and limited acceptance by clinical teams. Future research and policy efforts should focus on context-specific role definitions, including studies in more diverse and urban populations, training and qualification frameworks, as well as strategies for practical integration into routine health care in Germany.

## Supplementary material

10.2196/83073 Multimedia Appendix 1 Sociodemographic questionnaire for patients.

10.2196/83073 Multimedia Appendix 2 Interview guideline for patients.

10.2196/83073 Checklist 1 COREQ checklist.

## Abbreviations

COREQ: Consolidated Criteria for Reporting Qualitative Research
DHN: digital health navigator
DiGA: Digitale Gesundheitsanwendungen
DigiNavi: Digital Navigators for Acceptance and Competence Development with Mental Health Apps
DMHA: digital mental health app
DTx: digital therapeutics
GP: general practitioner
HCP: health care professional
WHO: World Health Organization
