# Supplementary material for: Expected Competencies and Personal Attributes of Digital Health Navigators to Support Digital Mental Health Care: Focus Group and Interview Study With Patients and Health Care Professionals
Source: JMIR Ment Health. 2026 Apr 23;13:e83073. doi: 10.2196/83073 (PMC13105428; doi:10.2196/83073)
Supplement: Multimedia Appendix 1 [file mental-v13-e83073-s001.docx]

| **DigiNavi** | **Sociodemographic Data** | | | | |
| --- | --- | --- | --- | --- | --- |
|  |  |  | **Study nmbr.:** |  | **BASELINE** |
|  | **ID:** |  | **Date:** |  |  |

**Name, First name:**

**1. Which gender do you identify with?**

☐ female

☐ male

☐ diverse

**2. How old are you now (in years, e.g. 58 years old)?**________________ years

**3. Which ethnic group do you belong to?**

☐ black ☐ PoC (Person of Color)* ☐ white
☐ other:____________________

**4. What is your highest level of education?**

☐ No school leaving certificate

☐ Primary/secondary school leaving certificate

☐ Secondary school leaving certificate/secondary school diploma

☐ A-levels

☐ Completed vocational training

☐ University degree

☐ other: ____________________

**5. Which of the following categories best describes your current employment status? (Multiple answers possible)**

☐ Fulltime

☐ Part-time up to 20 hours per week

☐ Part-time with less than 15 hours per week

☐ seeking employment

☐ retired

☐ currently unable to work

**6. Have you ever been or are you currently undergoing psychiatric/psychotherapeutic treatment?**

☐ yes

☐ no

☐ I am on the waiting list

**7.** **How would you rate your knowledge of digital health applications (DiGAs)?**

☐ No prior knowledge

☐ Very low

☐ Rather low

☐ Rather high

☐ Very high

**8. How many digital health applications have you been prescribed so far?**

☐ none

☐ 1 - 2

☐ 3 - 4

☐ 5 or more

**9. Please try to remember: When (in which year) were you first prescribed a digital health application (DiGA) or recommended one as part of your treatment?**

☐ 2024

☐ 2023

☐ 2022

☐ 2021

☐ never

**10. Do you use smartphone apps to improve your physical and mental well-being, e.g. a fitness app (e.g. yoga, Pilates app, etc.), a sleep or meditation app?**

☐ Yes

☐ No

**11. How confident or competent do you feel when using various types of digital applications (e.g. online banking, shopping, navigation, communication apps, health insurance apps)?**

☐ Very insecure

☐ Rather insecure

☐ Rather secure

☐ Very secure

**12. How do you usually proceed when you encounter technical problems with digital devices or software?**

☐ Seek help from others

☐ Try to solve it myself

☐ Use online help resources

☐ I rarely or never have technical problems

☐ Other, namely: __________________________________

**13. How would you rate your ability to learn and use new digital tools or software for your health needs or everyday tasks?**

☐ Very low

☐ Rather low

☐ Rather high

☐ Very high

**Thank you for participating!**
