# Supplementary material for: Expected Competencies and Personal Attributes of Digital Health Navigators to Support Digital Mental Health Care: Focus Group and Interview Study With Patients and Health Care Professionals
Source: JMIR Ment Health. 2026 Apr 23;13:e83073. doi: 10.2196/83073 (PMC13105428; doi:10.2196/83073)
Supplement: Multimedia Appendix 2 [file mental-v13-e83073-s002.docx]

Interview Guideline for Patients

**Introduction (5 minutes)**

- **Welcome:** Thank you for participating in this interview.
- **Introduction:** Brief introduction.
- **Purpose of the Interview:** We would like to understand your opinions and expectations regarding digital navigators. These professionals are intended to help you make better use of digital health applications (DiGAs).
  → We are particularly interested in your expectations, ideas, and any concerns you may have.
- **Confidentiality:** Your responses will remain anonymous.
- **Consent for Recording:** Is it okay if we record this conversation? (Consent agreement)
- **Any open questions?**

| **Topics** | **Open Introductory Question** | **Follow-up for clarification** |
| --- | --- | --- |
| Introduction to Digital Health Applications (DiGAs) **(10 minutes)** | | |
| Experience with DiGAs | Tell me about your experiences with DiGAs (digital health applications). | How have you come into contact with DiGAs?  What do you think about the idea of health apps being prescribed?  Have you already used DiGAs?  Can you imagine using a prescribed health app? What kind of support would be important for you?  If you haven´t used a DiGA yet, what would need to happen for you to try one? |

| Introduction to Digital Navigators **(15 minutes)** | | |
| --- | --- | --- |
| Expectations  (general) | Imagine a professional digital navigator helps you use a DiGA. What do you think about this kind of support? | How could a digital navigator assist you in using a DiGA?  What would be particularly important for you to feel comfortable receiving support from digital navigators? |
| Expectations (specific) | What would you expect from the guidance of a digital navigator when using DiGAs? | What kind of support would you like when using a DiGA for the first time?  How do you imagine regular contact with digital navigators?   - In what form? (email, phone call, in-person meeting) - How often? - Why would this type of contact be helpful for you?   Could the work of a digital navigator encourage you to use a DiGA regularly? If so, how and why? |
| Skills of Digital Navigators | What skills should a digital navigator have? What should they learn or be able to do? | What specific technical skills should digital navigators master?   - required knowledge of digital health applications   What social skills are important for digital navigators?   - What communication skills are necessary for effective interaction? - How important are empathy and patient-centered thinking?   What administrative skills should digital navigators possess?   - What knowledge about data protection and data security is necessary?   What medical knowledge should digital navigators have?   - What basic knowledge about mental illnesses and their treatment is required? - Should digital navigators be informed about the evidence and effectiveness of DiGAs? (if yes/no, why?) |
| Tasks | What specific tasks should digital navigators take on, or what kind of support would you like from them? | How could digital navigators assist you in using a DiGA? (selection, setup, explanation, engagement, etc.)  Should digital navigators provide:   - technical support (installation, troubleshooting) - personalized app recommendations - patient motivation - training in technical skills - help with data analysis and interpretation   → Why or why not? How should this support be provided? |
| **Part 0**   - So far, the usage of DiGAs and the integration of health apps into treatment has been limited in practice - There is often a lack of specific knowledge about these technologies, as DiGA-related content is not yet widely included in regular education and training programs - In the U.S., a new role in multidisciplinary medical teams has been introduced: the digital navigator - This is a specialist within the team who has deep expertise in DiGAs, personal experience with these technologies, and knowledge of best practices for their use - Providing technical support and promoting digital literacy among patients - Assisting with installation, explaining how apps work, and demonstrating their benefits - Helping patients safely integrate these technologies into their daily lives - Adapting the choice of technology to each patient´s specific medical and personal needs - Guiding patients through app use and supporting data interpretation and analysis | | |
| Support and Usefulness of Digital Navigators **(20 minutes)** | | |
| Opportunities | Could support from digital navigators be personally helpful for you? If yes/no, why? | What opportunities do you see in the use of digital navigators in general? |
| Opportunities | In which areas of your treatment could digital navigators be particularly useful? | How could digital navigators help you feel more confident in using DiGAs and managing your condition?  What specific challenges in your healthcare could digital navigators help address?  How could digital navigators assist you in reaching your health goals more quickly or efficiently? |
| Suitability for specific patient groups | Are there certain patient groups for whom you think digital navigators would be particularly useful or unsuitable? | What do you think about the use of digital navigators for older patients? Do you see any barriers?  What do you think about the use of digital navigators for patients with limited technical skills? Do you see any barriers?  What do you think about the use of digital navigators for patients with chronic illnesses? Do you see any barriers?  How suitable do you think digital navigators are for patients with severe mental illnesses? |
| Long-term benefits | What could a long-term collaboration with digital navigators look like for you? | What type of feedback or adjustments from digital navigators would be especially helpful for you?  Could continuous support from digital navigators improve your long-term health or treatment?  → If yes, why? |
|  | Could digital navigators help you better manage your condition?  → If yes, why and how? | How could digital navigators help you use DiGAs in a way that aligns with your individual health needs? |
|  | What would be the biggest benefit of working with digital navigators? | Could this support help you feel more comfortable and confident in using DiGAs?  What would need to happen for you to find working with digital navigators especially valuable?  What kind of support could digital navigators provide beyond just technical assistance? |
| Acceptance and Challenges of Digital Navigators **(10 minutes)** | | |
| Barriers | What difficulties or obstacles do you anticipate with the introduction or support of digital navigators? | What challenges might you face in using DiGAs in generell?  What challenges might arise when using DiGAs with support from digital navigators?  What could digital navigators do to ease your concerns or reservations about their role?  How could the support provided by digital navigators be improved to increase your acceptance? |
| Relationship between healthcare providers and patients | How could the introduction of digital navigators impact your relationship with your doctors or therapists? | How could collaboration between your healthcare providers and digital navigators work, particularly in relation to DiGAs?  Could digital navigators complement your treatment in a meaningful way without interfering with your existing care?  How could this be done? What measures could help?  How important is it to you that digital navigators regularly communicate with your healthcare providers?  How could the introduction of digital navigators improve or potentially affect the quality of your healthcare? |
| Digital and technical skills | Could working with digital navigators improve your digital skills?  → If yes, why?  Why is it important to you to strengthen your technical competence? |  |
| Perspective | What must be considered when implementing digital navigators for guided DiGA use? | |
| Conclusion | Is there anything else you would like to say or wish for on this topic? | |
